# Advice upon Medical Subjects

**Published:** 1870-01

**Authors:** 


### Domestic Medicine.

### Advice Upon Medical Subjects.

BY THE EDITOR.

CHAPPED HANDS AND LIPS.—Procure an ounce of pure glycerine, place a teaspoonful of it upon a plate, and with a case-knife rub into it ten grains of tannin and five grains of very finely pulverised camphor. Add this to the ounce of glycerine, shake it well, so as to thoroughly mix it, and anoint the hands and lips with it. It will heal the excoriated surface in two or three days.

RAT BITES.—We believe rat bites to be poisonous. If the wound made by the teeth of the animal does not bleed freely, inflammation and swelling of a serious character usually follows. Persons bitten by rats should apply to the nearest surgeon and have the wound freely cauterized, after which hot poultices should be applied.

CARBUNCLE.—Carbuncles are serious affairs, particularly when located about the neck. Cases of death are occasionally recorded from them. They should be lanced early and freely. Do not wait for them to "ripen." Before the pus becomes thin or approaches the surface, the surrounding parts become involved in the inflammation—the matter burrows under the muscle and so renders the case a serious one. Early lancing, and repeated hot bread-and-milk poultices are the remedies to be employed.

INJURIES FROM RUSTY NAILS.—Recently we have seen several severe injuries from stepping upon rusty nails. This often results in lock-jaw and death. One of the best applications we have ever used is bruised peach leaves, applied to the wound with a bandage. When the leaves cannot be had, bind the sole of the foot in warm tar. If the wound is a large one, it should be syringed out, once or twice, with a cup of warm water, in which twenty drops of the solution of carbolic acid has been dropped.

HOW TO CURE HOOPING COUGH.—Mothers will say *that* is worth knowing. But our information comes late in the season—still you can secure the remedy next summer and prepare it ready for use when occasion may require.

Take half an ounce of freshly gathered chestnut leaves, over which pour a pint of boiling water. Allow it to remain upon the stove—without boiling—one hour. Then add a pint of cold water and sufficient sugar to render it palatable. Strain and give it to the child to drink frequently during the day. It will cure hooping cough in two weeks.

INGROWING NAIL.—This very painful difficulty can be remedied in the following manner:

Place soft bees-wax about the nail, covering the entire toe except the *sore* portion and the nail. Then heat some lard in an iron spoon—make it very hot, and pour it upon the "proud flesh," immediately over the spot where the nail dips into the flesh. The wax shields the sound part from the heat, while no pain is produced in the diseased portion. This application will not only arrest the inflammation, but will cause the nail to curl up—giving permanent relief.

TAPE WORM.—It is one of the easiest things in the world to destroy a tape worm, yet people are in the habit of considering it one of the most difficult of undertakings. If any of our readers have such a tenant and desire his immediate ejectment; all that is necessary to do is to secure one quart of common pumpkin seeds. Pour over them two quarts of hot water, and allow them to stand for two days. While this is being done let the patient abstain from eating (for two days), and on the morning of the third day drink the emulsion from the seeds. At noon take one ounce of castor oil, and before night you will have the satisfaction of seeing the tape worm, head and all. There is nothing very disagreeable about this treatment, save the dieting, and the remedy never fails in its object.

WINTER SPORTS.—Now that winter sports have fairly commenced, see to it parents, that your children are allowed to engage in them freely. Be sure that they are properly clad—woollen next the skin—heavy boots and shoes—*and no scarfs about their necks.* Caution them not to sit upon the ice in adjusting their skates, always to throw off their shawls and overcoats when commencing the exercise, and to resume them again immediately upon resting or starting for home.

Free and jolly exercise for children, in the winter season, will cause more development of muscle, more strength and vigor to chest and lungs, than at any other season of the year. Do not keep them within doors for fear of the cold; clothe them warmly and let them run wild with skates, sleds, and shinney—its glorious sport and they know it. Let them run we say, you will have the satisfaction of seeing them "eat like little pigs," while their cheeks will rival the rose for redness, and their eyes will eclipse the diamond in brilliancy—these are symbols of health—let them run!

INFANT'S EYES.—Fond mammas and thoughtless nurses often expose the eyes of newly-born infants to the bright gas or lamp light. This is done with a view of examining its eyes, in order to learn their color, or to attract babie's attention to the fact that it is in possession of the sense of seeing. This is wrong—very wrong. From this cause alone, have we been called upon to prescribe for many cases of diseased eyes and blindness in infants.

Always have the gas light well shaded, so that it may not reach the bed of the infant. Never use a kerosene lamp in the room where the little one dwells. The gases escaping from the lamp will injure the eyes of an adult and much more so the sensitive ones of the infant. Where gas is not used, a sperm candle will give sufficient light for the lying-in-chamber.

CATARRH.—Do not use the "catarrh snuffs" that you see paraded in the advertising columns of almost every newspaper, now-a-days. Such applications to an inflammed or ulcerated surface can do no possible good but may do injury. If you desire to treat yourself, procure what is know as a "nasal douche"—you can find them in any first-class drug store, and will cost, with the bottle, from three to five dollars. Place in the bottle a pint of warm water, in which agitate from ten to twenty drops of the solution of carbolic acid, and pass it through the nostrils, as per directions accompanying the douche. Every alternate morning use a solution of salt instead of the acid—about half a teaspoonful of table salt to the pint of water. Ask your family physician to prescribe the proper amount of quinia and iron for you, and you will have the satisfaction of seeing all catarrhal symptoms rapidly disappear. But don't obstruct your nostrils with the nasty snuffs offered by patent medicine men.

USE OF THE PESSARY.—Most of these instruments do more harm than good, yet ladies persist in their use with the hope of obtaining relief from them, when absolute injury is being done the parts daily. Pessaries that are so constructed as to depend upon the walls of the vagina to hold them in place, are worse than useless. They not only promote ulceration and inflammation of the vagina, but are of little if *any* service in giving support to the womb. We therefore caution our lady readers against the use of all sorts of passeries, by whatever name known, that depend upon the walls of the vagina for support. The only uterine supporter that we have ever seen, that really gives any support to the uterus, is the one made by Dr. Babcock, of Illinois. This instrument is constructed upon proper principles, *having a direct support from an established base.* The cup is supported within the cavity, by means of a stem fastened to a belt about the waist. Such an instrument can be worn with safety and with some practical benefit.

CHILDREN'S EARS.—Scrofula, scarlet fever, and measles often cause discharges from the ears of children. The matter is usually very offensive, and if allowed to remain in the ears enfeables the child's health through its absorption into the circulation, and injures the hearing through ulceration of the drum and lining membrane. Mothers sometimes have an idea that such discharges should not be arrested—that if the discharge is "dried up," it will break out elsewhere, or that it "will go to the lungs." Nothing could be more absurd. The ears should be kept perfectly free from the matter by frequent applications of the ear-syringe, used with warm water in which a few drops of creasote or the solution of carbolic acid has been dropped. At night the ears should be cleansed and filled with the following:

Take—

Pure glycerine, 1 oz.

Tannin, 15 grains.

Place the glycerine on a dinner plate, which must be heated quite hot on a stove. Then rub the tannin into the glycerine thoroughly, with a case knife. Bottle the mixture and it is ready for use. Confine the mixture in the ears by means of cotton, which can be removed in the morning and the syringing again resorted to.

In old or chronic cases, some constitutional treatment will be necessary, for which a physician should be consulted.

WINTER CLOTHING.—We wish we were the happy father of a little daughter to match our two boys, and to teach people how to clothe a little girl decently and comfortably. We are sure we would'nt be foolish enough to dress her head fifteen dollar's worth—her neck twenty dollar's worth—her shoulders forty dollar's worth—and her hips and limbs *one* dollar's worth. Just look at a fashionable Mamma's little daughter! See her tripping along the street with a beautiful little hat upon her head, a costly velvet saque and set of furs upon her shoulders; while her slim little legs are encased in thin cotton hose, and a piece of fine cambric in the form of drawers. It's abomniable, is such a fashion. We would take delight, were we not a modest man, to see that little one's mamma out upon the street this cold morning, with skirts as short and limbs as thinly clad as those of her little daughter's. Our impression is that the child's wardrobe would be materially changed if the mother did not freeze to death before reaching home.

Heavy flannel drawers, thick woolen stockings, and warm overshoes is the necessary portion of a child's clothing at this season of the year. Over this you may place all the velvet, "fuss and feathers" that your purse can afford or your fancy suggest. But the child's health must not be sacrificed to fashion. Never allow your child to wear a scarf about the neck. The neck should be as bare as the face—except in riding. If the neck is bundled up with several yards of scarf, the child is certain to throw it off in the heat of play, or to run out doors without it. Severe and often fatal colds are contracted in this manner, as the pores of the skin are freely opened by the warmth of the scarf, and are suddenly closed by exposure to cold air, resulting in congestion of throat and lungs. Therefore never give your boy or girl a scarf.

Mothers, dress your little daughters with more regard to warmth and comfort than to fashion. If their little limbs don't look quite so neatly when covered with thick materials, their ruddy cheeks and sound lungs will more than compensate you for the seeming sacrifice you have made, and, then you will have the satisfaction too, of seeing your girls grow up to strong and vigorous womanhood.

